# 3D in vitro modelling of post-partum cardiovascular health reveals unique characteristics and signatures following hypertensive disorders in pregnancy

**DOI:** 10.1186/s13293-024-00672-6

**Published:** 2024-11-25

**Authors:** Clara Liu Chung Ming, Dillan Pienaar, Sahar Ghorbanpour, Hao Chen, Lynne Margaret Roberts, Louise Cole, Kristine C. McGrath, Matthew P. Padula, Amanda Henry, Carmine Gentile, Lana McClements

**Affiliations:** 1 School of Biomedical Engineering, Faculty of Engineering and Information Technology, University of Technology Sydney, Sydney, NSW Australia; 2 School of Life Sciences, Faculty of Science, University of Technology Sydney, Sydney, NSW Australia; 3 Department of Women’s and Children’s Health, St. George Hospital, Sydney, NSW Australia; 4 St George and Sutherland Clinical Campus, School of Clinical Medicine, University of New South Wales Medicine and Health, Sydney, NSW Australia; 5 Australian Institute of Microbiology and Infection, Faculty of Science, University of Technology Sydney, Sydney, NSW Australia; 6 Discipline of Women’s Health, School of Clinical Medicine, University of New South Wales Medicine and Health, Sydney, NSW Australia; 7 Present Address: Heart Research Institute, Sydney, Australia

**Keywords:** Pregnancy, Hypertensive disorders of pregnancy, Cardiovascular disease, Post-partum, Biomarkers, Proteomics

## Abstract

**Background:**

Hypertensive disorders of pregnancy (HDP) affect 2–8% of pregnancies and are associated postpartum with increased cardiovascular disease (CVD) risk, although mechanisms are poorly understood.

**Methods:**

Human induced pluripotent stem cells (iPSC)-derived cardiomyocytes, cardiac fibroblasts and coronary artery endothelial cells were cocultured to form cardiac spheroids (CSs) in collagen type-1 hydrogels containing 10% patient plasma collected five years postpartum [*n* = 5 per group: normotensive control, gestational hypertension (GH) and preeclampsia (PE)]. Plasma-treated CSs were assessed for cell viability and contractile function and subjected to immunofluorescence staining and imaging. A quantitative proteomic analysis of plasma samples was conducted (controls *n* = 21; GH *n* = 5; PE *n* = 12).

**Results:**

Contraction frequency (CF) was increased in PE-treated CSs (CF: 45.5 ± 3.4 contractions/minute, *p* < 0.001) and GH-treated CSs (CF: 45.7 ± 4.0 contractions/minute, *p* < 0.001), compared to controls (CF = 21.8 ± 2.6 contractions/min). Only PE-treated CSs presented significantly increased fractional shortening (FS) % (9.95 ± 1.8%, *p* < 0.05) compared to controls (3.7 ± 1.1%). GH-treated CSs showed a reduction in cell viability (*p* < 0.05) and an increase in α-SMA expression (*p* < 0.05). Proteomics analyses identified twenty differentially abundant proteins, with hemoglobin A2 being the only protein perturbed in both GH and PE versus control plasma (*p* < 0.05).

**Conclusions:**

The innovative patient-relevant CS platforms led to the discovery of biomarkers/targets linked to cell death signaling and cardiac remodeling in GH-induced CVD and vascular/endothelial cell dysfunction in PE-induced CVD.

**Supplementary Information:**

The online version contains supplementary material available at 10.1186/s13293-024-00672-6.

## Abstract (plain language summary)

Hypertensive disorders of pregnancy (HDP), including gestational hypertension (GH) and preeclampsia (PE), are a major cause of health problems and death for pregnant women. Women who experience HDPs have a much higher risk—up to 7 times greater—of developing cardiovascular disease (CVD) within 5–10 years after the affected pregnancy, and this risk persists for life. Although the link between HDP and future CVD is clear, the underlying causes are not well understood. This study developed a new 3D mini-heart model to investigate heart disease risk after HDP. We analyzed the patients’ plasma collected five years after pregnancy in our mini-heart model and compared GH and PE molecular profiles. Even when individuals appeared healthy based on routine heart and metabolic tests, our heart model detected early signs of heart dysfunction at the cellular and molecular levels. We found that using our model, the contractile mini-heart function was impaired post-PE or GH compared to the healthy control group. Meanwhile, post-GH the number of surviving cardiac cells were reduced and early signs of cardiac dysfunction were observed at the molecular level. We further analyzed the patients’ blood to understand the proteomic profile post-GH or -PE better. Our results showed that GH triggered proteins related to cell health, heart muscle function, and cell death, while PE caused problems with inflammation and proteins linked to blood vessel dysfunction. Understanding these processes could help develop targeted treatments for each condition and lead to personalized approaches to prevent and treat early-stage heart disease in this high-risk population of women post-HDP.

**Supplementary Information:**

The online version contains supplementary material available at 10.1186/s13293-024-00672-6.

## Highlights

- Gestational hypertension and preeclampsia appear to affect the heart five-years post-partum in differential manner.
- Gestational hypertension triggers systemic mechanisms related to cell health, heart muscle function, and cell death.
- Preeclampsia leads to perturbed inflammation and proteins linked to blood vessel dysfunction.
- Our 3D cardiac spheroid platform was able to detect early signs of cardiac dysfunction at the cellular and molecular levels post-gestational hypertension or preeclampsia despite the fact that the clinical cardiovascular and metabolic profiles of these individuals appeared healthy.

**Supplementary Information:**

The online version contains supplementary material available at 10.1186/s13293-024-00672-6.

## Introduction

Hypertensive disorders of pregnancy (HDP) affect 2–8% of all pregnancies​ and are a leading cause of mortality and morbidity in pregnancy ​ [1, 2]. HDP is characterized by pregnancy hypertension (defined as blood pressure ≥ 140/90 mmHg) including chronic hypertension (already present pre-pregnancy), gestational hypertension (GH) (new-onset at ≥ 20 weeks of gestation), or preeclampsia (PE), which may occur *de-novo* or superimposed on chronic hypertension and is characterized by evidence of maternal organ involvement and/or utero-placental dysfunction [1]​. Pathophysiological mechanisms of HDP remain incompletely elucidated; however, maladaptation of the maternal cardiovascular system appears to play a central role in GH, whereas poor placentation is a dominant link in PE ​ [2, 3]​. Moreover, individuals affected by HDP have an increased risk of developing cardiovascular disease (CVD), up to 7-fold higher than those who had normotensive pregnancies, within 5–10 years of affected pregnancy and continuing lifelong [4]. Even though the epidemiological link between HDP and future CVD is well-established, the mechanisms driving this association are poorly understood. This has impeded the development and implementation of effective monitoring and treatment strategies for women post-HDP, which are needed to ameliorate their CVD risk.

Heightened inflammation has been identified as one of the key mechanisms in HDP. Galectin-3 (Gal-3) has emerged as an important biomarker due to its key role in cardiac remodeling and heart failure alongside NT-proBNP and BNP, which was demonstrated in a recent meta-analysis ​ [5], and more recently in PE [6]. Another important marker of cardiac remodeling is alpha-smooth muscle actin (αSMA), highly expressed in activated cardiac myofibroblasts regulating cardiac fibrosis and subsequent heart failure [7]. Endothelial dysfunction is also important in the pathogenesis of both HDP and CVD, and can lead to high blood pressure, reduced vascular endothelial-cadherin (VE-cadherin) and angiogenesis [8, 9]. FK506-binding protein-like (FKBPL) is a critical molecule of developmental, physiological and pathological angiogenesis, a key determinant of CVD, and a predictive and diagnostic biomarker of PE [10–12]. All these molecules play a direct role in cardiac tissue hypertrophy and fibrosis, and may be potential targets for HDP-induced CVD [13, 14].

Nevertheless, the causes of HDP-induced cardiac dysfunction post-partum are still not well understood as current in vivo and in vitro models fail to recapitulate this complex disease [15]. In this study, we modeled, for the first time, HDP-induced CVD by using our clinically amenable in vitro cardiac spheroids (CS), capable of mimicking the molecular, cellular and extracellular features of the human heart microenvironment. CS have been shown to be an optimal tool for studying angiogenesis, contractile function and cell signaling regulating cardiovascular health [16–18]. They are generated by co-culturing human induced-pluripotent stem cells-derived cardiomyocytes (iCMs), human coronary artery endothelial cells (HCAECs) and human cardiac fibroblasts (HCFs) at ratios approximating the ones found in the human heart [16, 19]. In a recent study, we demonstrated that CSs generated from HCFs and HCAECs and treated with plasma from women with early-onset preeclampsia (EOPE; diagnosed before 34 weeks of gestation) or late-onset preeclampsia (LOPE; diagnosed from 34 weeks of gestation) can be used to study changes in angiogenesis, such as the increase in an anti-angiogenic protein, FKBPL, in the context of LOPE [20].

Here, we demonstrate, for the first time, that patient-relevant 3D CSs is an effective platform to model HDP-induced CVD post-partum, facilitating identification of early cardiovascular changes. We show distinct cellular and molecular changes five-year post GH or PE, compared to healthy pregnancy, despite no differences in cardiovascular and metabolic clinical characteristics. Both GH and PE plasma treatment led to aberrant cardiac function as measured by the CS contractility assay. Interestingly, GH-relevant CSs show early signs of cardiac cell death and fibrosis due to increased α-SMA expression, whereas PE-relevant CSs were characterized by heightened inflammation due to higher Gal-3 expression. Finally, to better understand the mechanism within the secretome driving these cellular and molecular changes with CSs, we conducted comprehensive unbiased and untargeted proteomics analysis and identified twenty differentially abundant proteins between these three groups that could be viable therapeutic targets or biomarkers. The vast majority of proteins identified show the differences between PE and GH, representative of unique proteome profiles five-year post-GH or PE, which were characterized by disrupted cell death signaling and cardiac remodeling or vascular and endothelial dysfunction, respectively.

## Methodology

### Reagents

Fibronectin from bovine plasma was purchased from Sigma-Aldrich (Missouri, USA). Cardiomyocytes iCell Plating Medium and iCell Maintenance Culture Medium were purchased from Cellular Dynamics (Wisconsin, USA). L-glutamine solution, penicillin-streptomycin and TrypLE were purchased from Thermo Fisher Scientific (Massachusetts, USA).

### Plasma isolation from patients

Peripheral blood was collected from patients as part of the P4 study presenting five-years after PE (*n* = 11), GH (*n* = 5) and normotensive pregnancy (*n* = 21) [21]. Blood was collected in Lithium Heparin Plasma tubes and centrifuged at 3000*g* × 10 min to collect plasma. The plasma from each participant was aliquoted and stored at − 80 °C. The study was conducted in accordance with the Declaration of Helsinki and approved by both South Eastern Sydney Local Health District and University of Technology Sydney, Human Ethics Committees.

### Cell cultures

Cells were cultured according to our previously established protocols [16, 17]. Briefly, HCAECs were cultured in MesoEndo Culture Media (Sigma-Aldrich, Missouri, USA), whereas HCFs were cultured in Cardiac Fibroblast Growth Media (Sigma-Aldrich, Missouri, USA) both supplemented with 1% penicillin streptomycin and 1% L-glutamine. Both HCAECs and HCFs were isolated from both male and female donors. Media was changed every 2–3 days and cells cultured until 70–90% confluency before being passaged using TrypLE (passage 6 to 8 for HCAECs and passage 3–5 for HCFs). A cryovial of iCMs (5 × 10^6^ cells, iCells^2^, from a female Caucasian donor; Cellular Dynamics, Wisconsin, USA) was thawed and transferred to a fibronectin-precoated T-75 tissue flask and incubated for 20 h with iCell Plating Media at 37^o^C and 5% CO_2_, following the manufacturer’s guidelines. After 20 h, media was replaced with iCell Maintenance Media and after 72 h, iCMs were detached with TrypLE for CS formation.

### Cardiac spheroid (CS) formation

Cardiac spheroids were generated according to our previously established protocols [16, 17]. Briefly, prior to CS formation, agarose powder (Sigma-Aldrich, Missouri, USA) and 3D micro moulds (Sigma-Aldrich, Missouri, USA) were sterilized. Agarose powder (1%) was reconstituted in phosphate buffered saline (PBS) solution, then heated for a few seconds to allow proper dissolution of the powder, and finally pipetted into 3D micromoulds to set and form moulds for spheroids. Solidified moulds were submerged in culture media and incubated at 37^o^C and 5% CO_2_, following the manufacturer’s guidelines.

CSs were formed as per our previously established protocols [19, 22]. Briefly, HCAECs, HCFs and iCMs were detached from their respective flasks with TrypLE, counted and mixed in a ratio of 2:1:1 (iCMs: HCAECs: HCFs). Cell pellets were resuspended in CS culture media (iCM: HCAEC: HCF media at a ratio of 2:1:1, respectively). Cell suspension (190 µl) was added to the inner cavity of the agarose moulds and incubated at 37 ^o^C and 5% CO_2_ with daily media changes. Following 48 h, CS were collected and carefully transferred to a 96-well plate; (four to eight CSs were used per patient plasma and a minimum of five CSs per well). Media was replaced with a solution containing 1:1 media: collagen 1 rat tail (Merck, Massachusetts, USA) and the whole plate was moved to an incubator at 37 ^o^C and 5% CO_2_ overnight.

### Plasma treatment of CSs

Patient-derived plasma samples were added onto wells containing CSs in collagen hydrogels for 96 h and incubated at 37 ^o^C and 5% CO_2_. Plasma samples from age and BMI-matched PE, GH and normotensive control groups (*n* = 5 per group) were added as 10% of the total volume. A media change occurred at 48 h to ensure cells remained viable.

### Live and dead assay

CSs embedded in collagen were washed twice with Dulbecco′s Phosphate Buffered Saline. Live/Dead^®^ Viability/Cytotoxicity Kit for mammalian cells and NucBlue^®^ Live ReadyProbes^®^ Reagent (Hoechst 33342) (Invitrogen, Massachusetts, USA) were used according to the manufacturer’s instructions. The cell viability was determined by quantifying live to dead cell ratio using an EVOS M7000 Imaging System (Invitrogen, Massachusetts, USA). Changes in viability were reported as fold changes compared to the mean of values in control cultures (equal to 1).

### Contractile activity assay

Following our previously published protocol [19], the contractile activity of CSs was evaluated using videos of contracting CSs acquired with a Nikon Eclipse TiE2- widefield fluorescence microscope. Videos were used to measure fractional shortening percentage (FS%, % of difference between contracted and relaxed CS diameter, Supplementary videos 1–3) and frequency of contractions (number of contractions per minute).

### Immunolabeling and confocal imaging

Media were removed from each well, and CSs were fixed with 10% formalin (Sigma-Aldrich, Missouri, USA) for one hour at room temperature. Post incubation, cells were washed three times with phosphate buffer saline containing 1% sodium azide (PBSA), permeabilized with 0.2% Triton X-100 for 30 min and then blocked in 3% bovine serum albumin (BSA)/PBSA overnight at 4 °C on a rocking plate. CSs were then probed with Galectin-3 (1:100, Abcam, Cambridge, UK), FKBPL (1:100, Proteintech, Rosemont, USA), CD31 (1:10, BD Biosciences, Franklin Lakes, USA), VE-cadherin (1:100, Abcam, Cambridge, UK), αSMA (1:100, Abcam, Cambridge, UK) primary antibodies and incubated at 4 °C overnight. Following washing, goat anti-rabbit IgG H&L (Alexa Fluor^®^ 488, Abcam, Cambridge, UK) and goat anti-mouse IgG H&L (Alexa Fluor^®^ 594, Abcam, Cambridge, UK), and Cy5-Donkey anti-mouse (1:142, Jackson ImmunoResearch, Pennsylvania, USA) secondary antibodies were added and incubated at 4 °C overnight. CSs were then incubated with Vimentin (1:250, Abcam, Cambridge, UK) and Troponin C (1:10, Santa Cruz, California, USA) primary-conjugated antibodies and DAPI (10 µg/ml, Invitrogen, Massachusetts, USA) at 4 °C overnight. Finally, CSs were washed three times with PBSA and stored at 4 °C.

Confocal fluorescence images were acquired with Leica Stellaris confocal, employing a 20× objective with a numerical aperture (NA) of 1.45 and Nyquist sampling. Z stacks were obtained with 0.9 μm optical slices, utilizing 1 AU pinhole to achieve high-resolution imaging. These confocal images were used as representative high-resolution visuals. For quantitative analysis of protein expression, widefield fluorescence images were acquired using Nikon Ti2-E, with a 20× objective, NA 0.75 and a long working distance of 2300 μm (Supplementary Figs. 1–3). Widefield images were then clarified using NIS-Elements Clarify.ai [23]. The fluorescent signal intensity, an indicator of protein expression, was quantitatively analyzed using ImageJ software (NIH, USA, version 2.1.0) on maximum-intensity projection images. The analysis involved ≥ 3 CSs per condition and the fluorescent intensity was subsequently normalized to the nuclear count.

### Preparation of plasma for proteomics analysis

Using an Acquity M-class nanoLC system (Waters, USA), 2 µL of the sample was loaded with MS Solvent A (0.1% Formic Acid) onto a HSS T3 column (Waters; 300 μm x 150 mm) heated to 50 °C at 5 µl/min. Peptides were eluted from the column and into the source of a Synapt XS mass spectrometer (Waters Instruments) using the following program: 1–40% MS solvent B (100% Acetonitrile) over 46 min, 40–85% MS solvent B over 1 min, 85% MS solvent B for 2 min, 85–100% for 1 min. The eluting peptides were ionized at 3000 V at a fixed Cone Voltage of 20 V. A UDMSE experiment was performed in positive mode with the Analyser Mode set to Resolution and Dynamic Range set to Extended. Peptide ions were first separated by Travelling Wave Ion Mobility Spectrometry (TWIMS) at a Transfer Wave Velocity of 155 m/s and applying a Charge State/Drift Time Stripping Rule file to remove 1 + ions during the Low CID Energy scan before being subjected to alternating low energy (6 eV) and high energy Collision Induced Dissociation (CID) with an accumulation time of 0.4 s for each scan type. High Energy CID was performed in the Transfer cell using a Look Up Table adapted from Distler et al. [24]. TOF scans were performed over the mass range of 50–1500 m/z. Total run time per sample was 60 min. The MS/MS data files were searched using the Ion Accounting method in Progenesis QI for Proteomics against the UniProt Human Proteome database (downloaded 01/03/2021) as previously described [5, 25].

### Statistical analysis

Images were analyzed with ImageJ for cell viability, contraction frequency, fractional shortening, and protein expression measurements within CSs. CS protein expressions were calculated as the Corrected Total Cell Fluorescence (CTCF) and represented as a fold change compared to the control group. Statistical analysis was performed using GraphPad Prism 9 software. Normality testing was performed using the Shapiro-Wilks test before parametric or non-parametric tests were employed based on the normal distribution of the data. If normally distributed, the data was analyzed using a two-tailed unpaired t-test or one-way ANOVA with post-hoc multiple comparison tests. For non-normally distributed data, Mann-Whitney or Kruskal-Wallis were used where appropriate. P-value < 0.05 was considered statistically significant.

## Results

### GH-derived plasma reduces cell viability in CSs

**Fig. 1:**
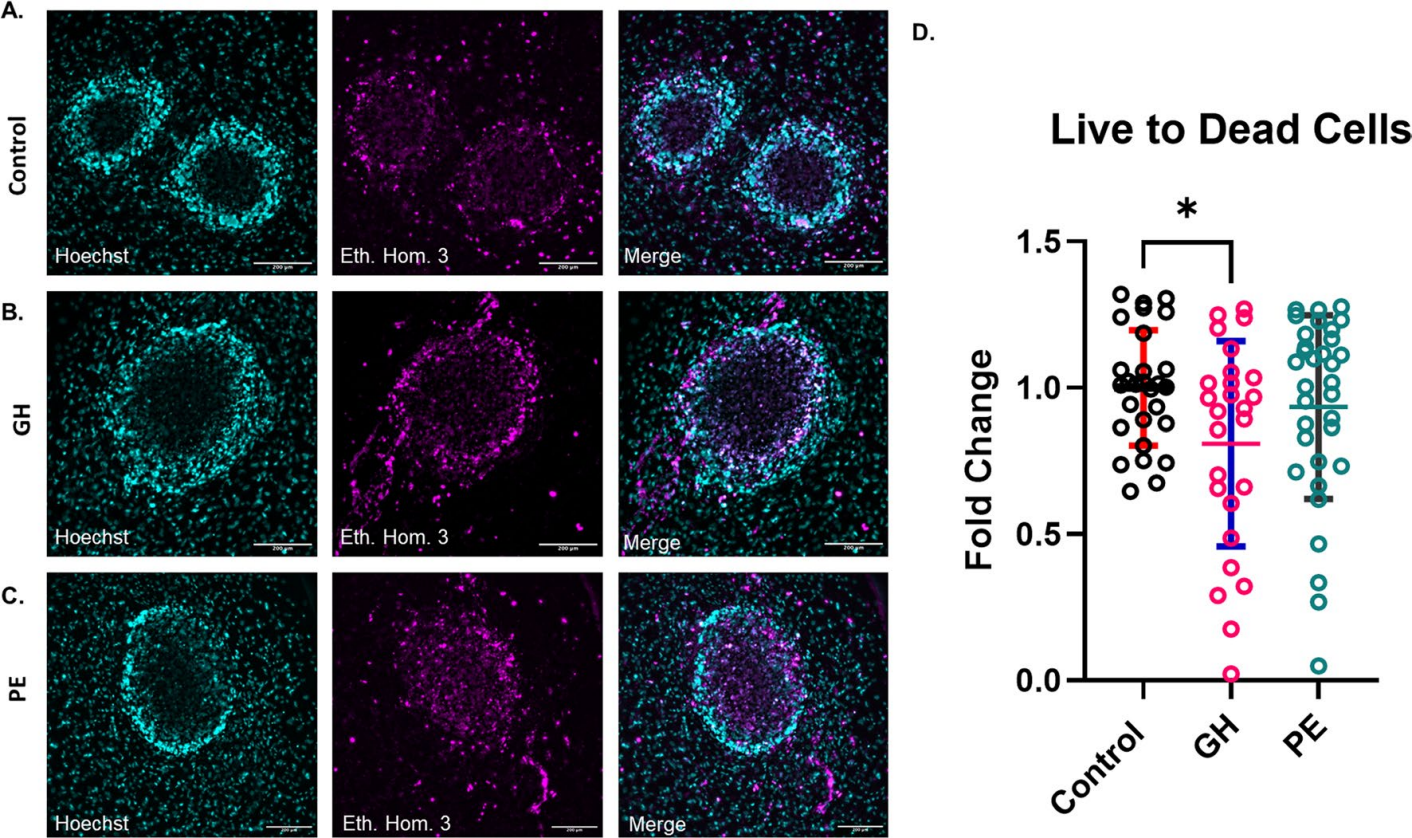
Plasma-derived from women with HDP reduces the cell viability of CSs. **A**-**C** Representative collapsed Z-stacks images of all and dead cells within CSs. The percentage of live cells remaining following exposure to human plasma from women with HDP was quantified for **A**) Control (normotensive plasma). **B** GH plasma. **C** PE plasma. 10% of normotensive, GH or PE plasma are added to CSs plated in collagen hydrogel, respectively. After 96 h, CSs were stained with Hoechst for nuclei stain, and ethidium homodimer for dead cells. Scale bar equals 200 µM. **D** Statistical analysis of the fold change in live versus dead cells comparing all three sample groups: controls, GH and PE. Data represented as Mean ± SD; *n* = 5 patients per group; P-value was calculated using one-way ANOVA with Tukey’s multiple comparisons test; * *p* < 0.05

To assess the effects of normotensive, GH or PE plasma on CS viability, the percentage of live cells was quantified by counting dead cells stained with ethidium homodimer and by normalizing against total cells stained with Hoechst (Fig. 1). There was a significant but modest decrease of 19.1 ± 8.0%, (*p* < 0.05) live versus dead cell ratio for GH-treated CSs compared to control cultures. In contrast, the reduction in the live versus dead ratio for PE-treated CSs was not statistically significant (6.5 ± 7.5%).

### Contraction frequency and fractional shortening % are increased in HDP-plasma treated CSs

**Fig. 2:**
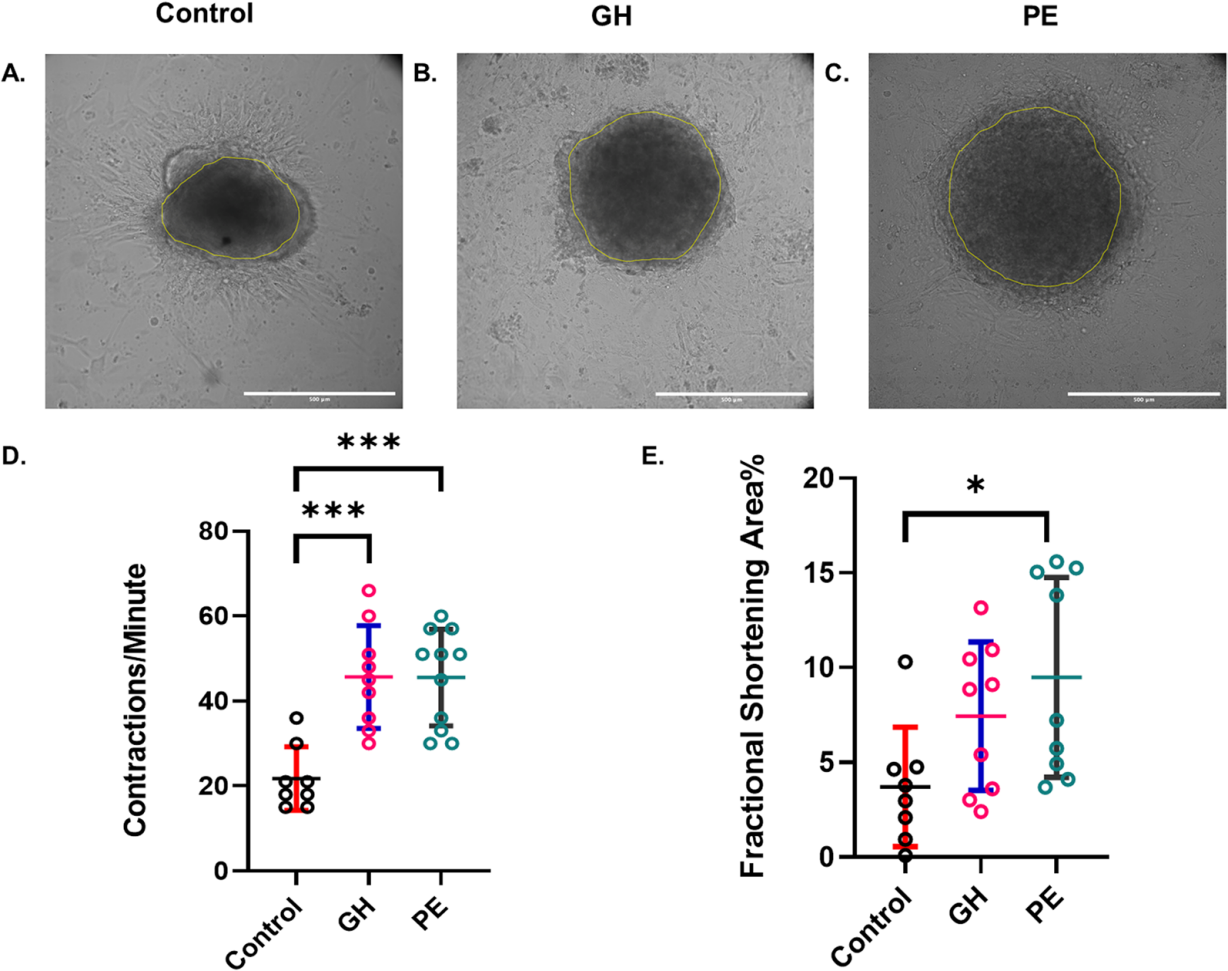
HDP-derived plasma impairs contractile function in CSs. **A**-**C** representative images from videos of contracting control- (**A**), GH- (**B**) and PE- (**C**) treated CSs, where yellow outlines the area of the cardiac spheroid during the phase of contraction in each spheroid. Scale bar 500 μm. (D-E) Statistical analyses of contraction frequency (**D**) and fractional shortening % (**E**) in CS. 4–8 CSs per group from 1–3 patients per group; The number of CSs per group: control (*n* = 8), GH (*n* = 9), PE (*n* = 9–11); P-value was calculated using one-way ANOVA post-hoc analysis. Data represented as Mean ± SD; *n* = 1–3 patients per group; *n* ≥ 8; P-value was determined using one-way post-hoc analysis; * *p* < 0.05, *** *p* < 0.001

To evaluate the effects of normotensive, GH and PE plasma on CS functionality, we measured the fractional shortening % (FS%) and contraction frequency of CSs following exposure to plasma for 96 h. Similar to our previous studies [26, 27], this was done by recording the time frame of each CS for 30 s using Nikon Ti2 microscope. As shown in Fig. 2, contraction frequency was significantly increased in PE-treated CSs (CF: 45.5 ± 11.0 contractions/minute, *p* < 0.001, Supplementary video 1) and GH-treated CSs (CF: 45.7 ± 12.1 contractions/min, *p* < 0.001, Supplementary video 2), compared to controls (CF: 21.7 ± 7.5, Supplementary video 3). Whilst HDP plasma increased the fractional shortening %, this was statistically significant for PE-relevant CSs only (9.5 ± 5.3%, *P* < 0.03) compared to controls (3.7 ± 3.1%). Altogether, our results showed that GH-derived plasma impairs both cell viability and contractile function in CSs, while PE-derived plasma only impairs the contractile function of CSs (Figs. 1 , 2).

### Key cardiac cell populations do not change following treatment with HDP-derived plasma

**Fig. 3:**
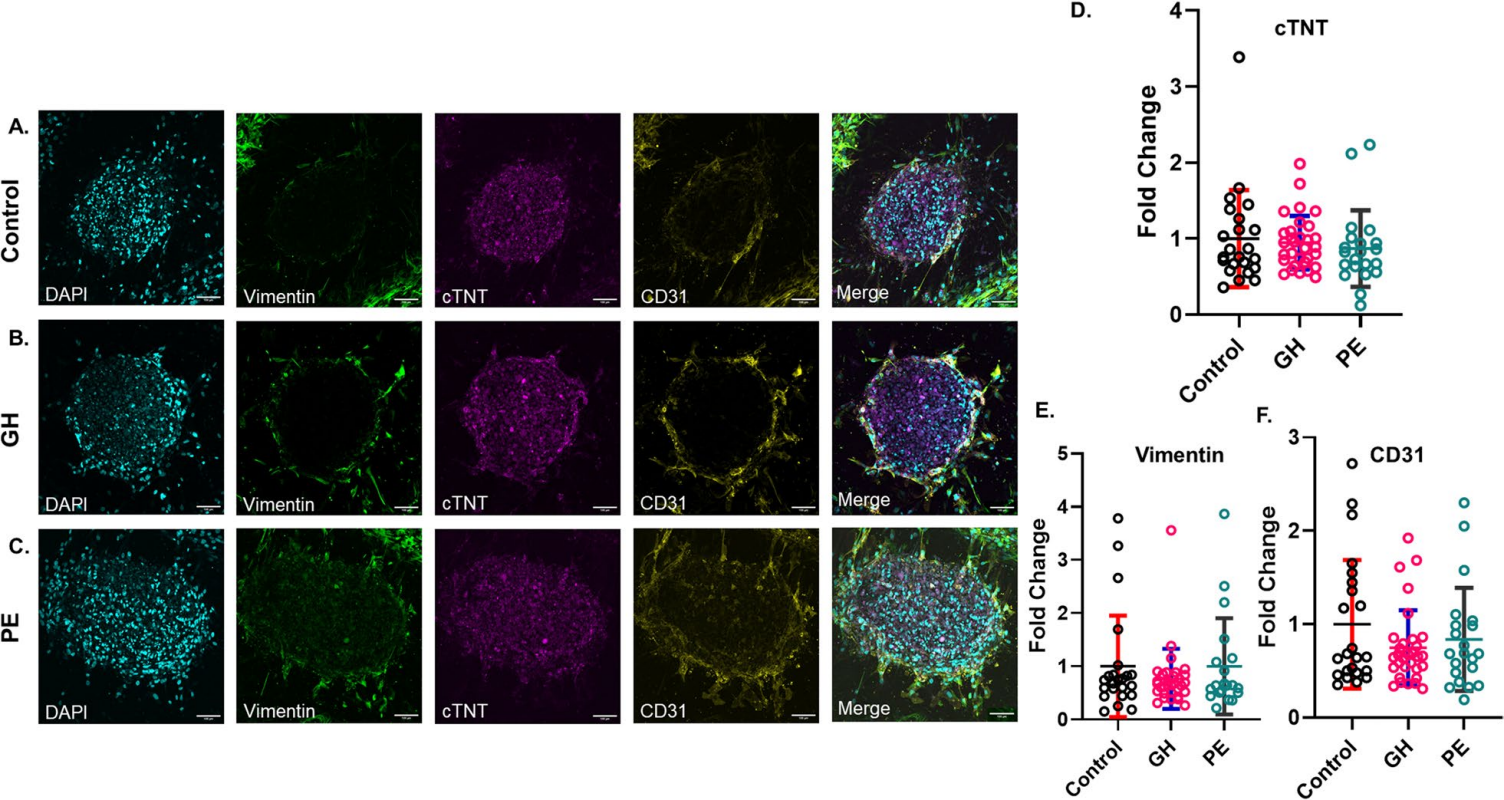
Treatment with HDP plasma does not change the numbers of cardiomyocytes, cardiac endothelial cells and cardiac fibroblasts in CSs. **A**-**C** Representative maximum-intensity projection confocal images of CSs stained for the three cell markers. CSs were exposed to 10% plasma (**A**) normotensive (control), (**B**) GH, and (**C**) PE, for 96 h, fixed and stained with antibodies against cardiomyocytes (cTNT, magenta), endothelial cells (CD31, yellow) and fibroblasts (vimentin, green). Nuclei are stained with DAPI stain (cyan). Confocal images are shown for representation only; widefield images were used for quantification of protein expression. Scale bar 100 μm. **D**-**F** Statistical analysis of the fold change in protein expression of vimentin (**D**), cTNT (**E**) and CD31 (**F**) for normotensive, GH and PE CSs, normalized to control. Data represented as Mean ± SD; *n* = 5 patients per group; The number of CSs per group: control (*n* = 23), GH (*n* = 31–32) and PE (*n* = 20–21); Kruskal-Wallis test witht Dunn’s multiple comparison test

To evaluate if HDP-derived plasma altered the overall cardiac cell population within CSs, we performed immunofluorescence staining to semi-quantitatively determine the expression of specific cell markers identifying three key cardiac cell types between the three groups (Fig. 3). CSs were stained with antibodies against cardiac troponin (cTNT) for iCMs, CD31 for HCAECs and vimentin for HCFs (Supplementary Fig. 1). The addition of HDP-derived plasma did not affect any of the cell types across normotensive, GH and PE groups. This suggests that the secretome present within plasma from women five years post GH or PE does not specifically affect a particular cardiac cell type.

### Pro-fibrotic α-SMA is increased in the presence of GH plasma, Gal-3 is increased in PE-treated CSs

**Fig. 4:**
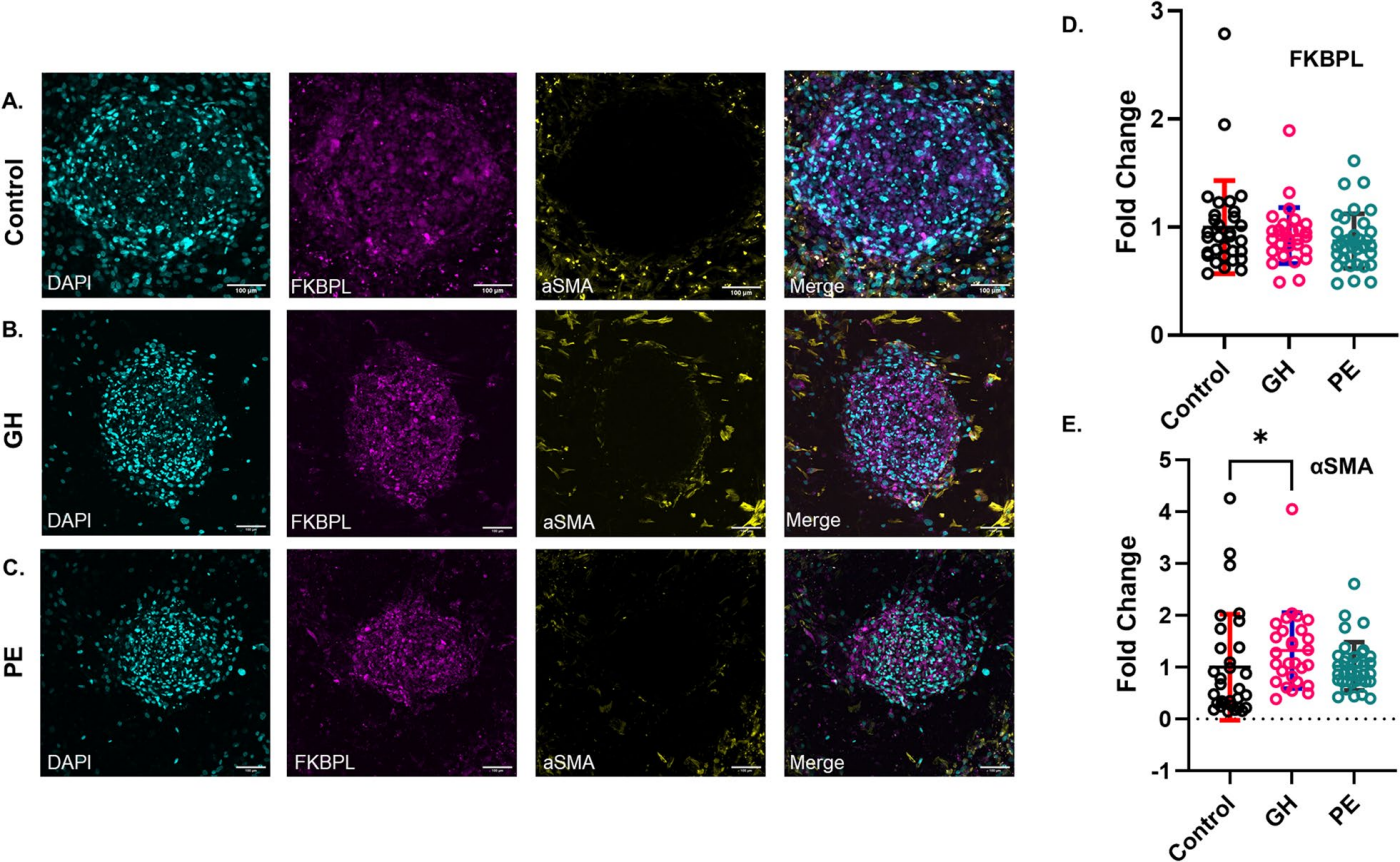
HDP plasma treatment increases α-SMA in CSs. **A**-**C** Maximum-intensity projection images from confocal Z-stacks of CSs exposed to normotensive (control) (**A**), GH (**B**) and PE (**C**) plasma for 96 h. Samples were stained with DAPI (cyan) for nuclei stain, as well as antibodies against FKBPL (magenta) and α-SMA (yellow). Confocal images are presented for visual representation only, while widefield images were utilized for the quantification of protein expression. Scale bar 100 μm. **D** Statistical analysis of the fold change of protein expression of FKBPL for control, GH and PE groups, normalized to control. **E** Statistical analysis of the fold change of protein expression of α-SMA for control, GH and PE groups, normalized to control. The number of CSs per group: control (n=31), GH (n=28-29) and PE (n=36). Data represented as Mean ± SD; *n* = 5 patients per group; P-value was determined using Kruskal-Wallis test with Dunn’s multiple comparison test; *p < 0.05

To evaluate if HDP plasma played any role in angiogenesis and fibrosis in CSs, we measured the changes in FKBPL and α-SMA protein expression, respectively (Fig. 4A-C, Supplementary Fig. 2). Our statistical analysis of protein abundance in Fig. 4D showed no significant changes in FKBPL protein abundance in GH and PE groups compared to controls. Furthermore, we measured α-SMA protein abundance, given its important role in cardiac remodelling and fibrosis [28]. The addition of GH plasma induced a significant increase in α-SMA protein abundance compared to the control (*p* = 0.01), whereas this was not statistically significant for the PE group (Fig. 4E).

**Fig. 5:**
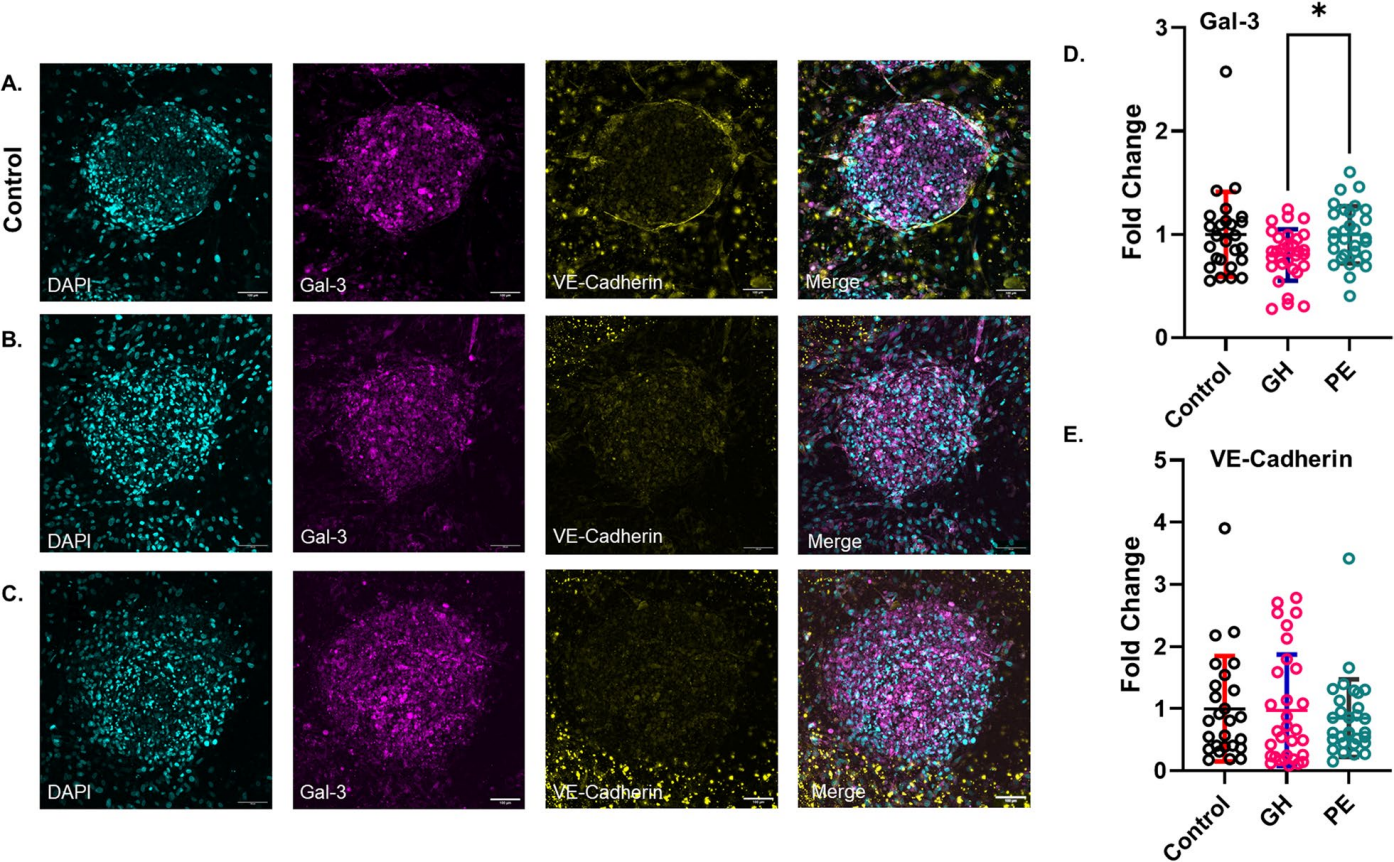
Gal-3 is increased in PE-treated CSs, and no changes in VE-Cadherin among groups were shown. **A**-**C** Maximum-intensity projection images from confocal Z-stacks of CSs treated with normotensive (control) (**A**), GH (**B**) or PE (**C**) plasma for 96 h. Samples were stained with DAPI (cyan) for nuclei stain, as well as antibodies against Gal-3 (magenta) and VE-Cadherin (yellow). Confocal images are shown for illustration purposes only, while widefield images were used for quantitative analysis of protein expression. Scale bar 100 μm. **D** Statistical analysis of the fold change of protein expression of Gal-3 for control, GH and PE groups, normalized to control. **E** Statistical analysis of the fold change of protein expression of VE-Cadherin for control, GH and PE groups, normalized to control. Data represented as Mean ± SD; *n* = 5 patients per group; The number of CSs per group: control (*n* = 26), GH (*n* = 31) and PE (*n* = 30); P-value was calculated using Kruskal-Wallis test with Dunn’s multiple comparison test; *p<0.05

Moreover, we examined the abundance of inflammatory and vascular stability markers, Gal-3 and VE-Cadherin (Supplementary Fig. 3), respectively, after HDP plasma treatment of CSs. Gal-3 protein abundance was increased (*p* = 0.04) in the PE-treated CS group compared to the GH-treated CSs but not in the control group (Fig. 5D). We did not detect any changes in VE-cadherin protein abundance across the three groups (Fig. 5E). Altogether, our results from the markers studied support an enhanced inflammatory response in PE plasma compared to GH (Figs. 4 and 5).

### Plasma proteomics analysis

In order to identify the mechanisms driving some of the changes observed in CSs functional, inflammatory and fibrotic measurements, differences in the secretome proteome profile across the different HDP groups and normotensive controls were performed using untargeted proteomics analyses with all available P4 substudy samples [21]. In terms of cardiovascular and metabolic clinical characteristics measured at 5 years postpartum, there were no significant differences between the three groups in the most relevant parameters, including age, body mass index (BMI), systolic blood pressure (SBP), diastolic blood pressure (DBP), blood glucose levels, insulin, HOMA-IR, HbA1c, eGFR, cholesterol, triglycerides, HDL, LDL and smoking status (Table 1). The vast majority of participants were Caucasians (*n* = 36), one South American, two Asian and one Aboriginal/Torres Strait Islander. None of the participants had any medical history that suggests an increased risk of CVD. The blood pressure and metabolic data was measured at the five-year follow up visit. The only difference noted was in gestational age at delivery between the control and PE group (*p* = 0.008). The PE group had a mixture of EOPE and LOPE; 3/11 participants had presented with EOPE.

**Table 1:** Clinical characteristics of P4 participants subgroups

Characteristics	Control (*n* = 21)			GH (*n* = 5)			PE (*n* = 11)			*p*-value
Age (Years)	39.6 ± 4.3			38.3 ± 5.0			37.0 ± 4.3			0.2
Time post-pregnancy (months)	61.6 ± 1.3			61.6 ± 1.4			61.9 ± 1.6			0.87
Gestational age at delivery (weeks)	39.6 ± 1.7			38.4 ± 0.4			36.9 ± 3.3**			0.01
Gestational age at diagnosis (weeks)	n/a			36.80 ± 2.2			35.70 ± 4.00			0.58
Fetal sex (% female)	48%			20%			55%			n/a
Severe hypertension (%)	n/a			20%			45%			n/a
Magnesium sulfate infusion (%)	n/a			0%			27%			n/a
Eclamptic seizures (%)	n/a			0%			9%			n/a
Hydralazine infusion (%)	n/a			0%			9%			n/a
BMI (kg/m^2^)	26.4 ± 6.0			32.2 ± 8.9			26.6 ± 5.1			0.17
Average SBP (mmHg)	109.4 ± 25.7			118.7 ± 13.1			113.6 ± 8.3			0.14
Average DBP (mmHg)	71.6 ± 18			76.3 ± 9.8			75.4 ± 8.2			0.38
Glucose (mmol/L)	4.7 ± 0.6			4.6 ± 0.4			4.5 ± 0.5			0.61
Insulin (mU/L)	8.7 ± 5.8			13.1 ± 10.2			12.6 ± 11.0			0.34
HOMA-IR	1.8 ± 1.2			2.7 ± 2.1			2.7 ± 2.5			0.35
HbA1c (%)	5.2 ± 0.3			5.3 ± 0.2			5.2 ± 0.3			0.8
eGFR (mL/min)	98.7 ± 4.4			98.0 ± 4.5			96.5 ± 6.1			0.5
Cholesterol (mmol/L)	4.7 ± 0.7			4.9 ± 0.5			4.6 ± 0.9			0.67
Triglycerides (mmol/L)	1.0 ± 0.7			1.1 ± 0.3			1.0 ± 0.6			0.94
HDL (mmol/L)	1.5 ± 0.3			1.4 ± 0.3			1.6 ± 0.4			0.74
LDL (mmol/L)	2.8 ± 0.6			3.0 ± 0.6			2.5 ± 0.7			0.39
Smoking %	Yes (0%)	No (76%)	Ex (23%)	Yes (0%)	No (80%)	Ex (20%)	Yes (0%)	No (64%)	Ex (36%)	n/a

Data presented as mean ± SD; Ordinary one-way ANOVA

*BMI* body mass index,* SBP* systolic blood pressure,* DBP* diastolic blood pressure,* HOMA-IR* homeostatic Model Assessment for Insulin Resistance,* HbA1c* glycated haemoglobin,* eGFR* estimated glomerular filtration rate,* HDL* high density lipoprotein,* LDL* low density lipoprotein

**p<0.01 (compared to control)

**Fig. 6:**
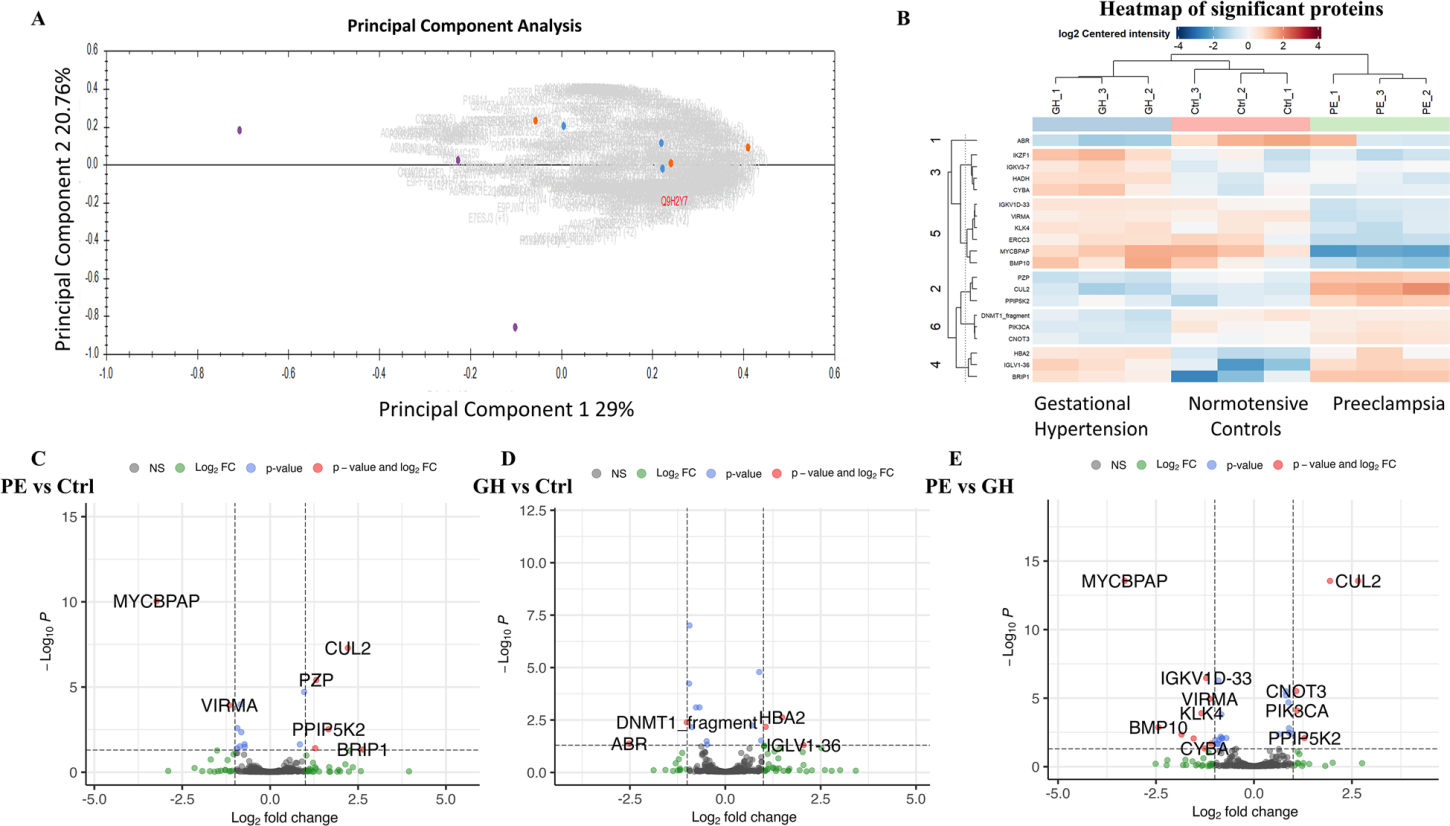
Differential expression of grouped patient plasma following untargeted proteomics analyses. Grouped samples were measured in triplicates to account for anomalies. (**A)** Principal component analysis (PCA) plot of grouped proteomic data for control (purple dots), GH (blue dots) and PE (orange dots) generated using Progenesis. (**B)** Multigroup heatmap with hierarchical clustering dendrogram of proteomic data levels across control, GH and PE groups. Volcano plots of proteomic data for (**C**) PE vs. normotensive (**D**) GH versus normotensive (**E**) PE vs. GH. Significant proteins were defined as Benjamini–Hochberg adjusted p-value < 0.05

Our quantitative label-free proteomic analysis of non-depleted pooled plasma samples was conducted by measuring the relative abundance of tryptic peptides using DDA mass spectrometry [5, 25, 28]. This detected 573 proteins across the grouped samples with a minimal percentage of missing values. The heterogeneity of grouped plasma samples was demonstrated through the hierarchical clustering of sample triplicates in the multigroup heat map and principal component analysis (PCA) plot (Fig. 6A). The lack of clustering is quite common in proteomics, particularly with patient plasma samples, due to high inter-patient variation.

Subsequently, differential abundance (DE) analysis was performed by three individual comparisons, PE versus control, GH versus control group and PE versus GH. Post-DE analysis, 20 unique proteins were identified in at least one of the group comparisons, with some proteins being significant in multiple comparisons. Across groups, 8 proteins were differentially abundant in PE versus control group, 6 proteins in GH versus control group and 15 proteins in PE versus GH (Table 2). Data are available via ProteomeXchange with identifier PXD051288.

**Table 2:** Multiple group comparisons for differential expression of plasma proteins

Protein	Adj. *p*-value	Ratio
*Preeclampsia versus control*		
BRIP1 (BRCA1 interacting helicase 1)	0.046	2.64
CUL2 (Cullin-2)	5.11E–08	2.21
PPIP5K2 (Diphosphoinositol Pentakisphosphate Kinase 2)	0.003	1.66
PZP (Pregnancy zone protein)	3.87E–06	1.31
HbA2 (Hemoglobin A2)	0.039	1.28
IGKV1D-33 (Immunoglobulin kappa variable 1D-33)	0.00258	− 0.934
VIRMA (Vir Like M6A Methyltransferase Associated)	0.0001	− 1.16
MYCBPAP (MYCBP Associated Protein)	8.95E–11	− 3.23
*Gestational hypertension versus control*		
IGLV1-36 (Immunoglobulin Lambda Variable 1–36)	0.0498	2.05
HbA2 (Hemoglobin A2)	0.002	1.49
IGKV3-7 (Immunoglobulin Kappa Variable 3–7 (Non-Functional))	0.007	1.06
PIK3CA (phosphatidylinositol-4,5-bisphosphate 3-kinase catalytic subunit alpha)	0.007	− 0.871
DNMT1_fragment (DNA-methyltransferase 1)	0.004	− 1.01
ABR (ABR Activator Of RhoGEF And GTPase)	0.042	− 2.52
*Preeclampsia versus gestational hypertension*		
CUL2 (Cullin-2)	2.81E–14	2.66
PZP (Pregnancy zone protein)	2.81E–14	1.94
PPIP5K2 (Diphosphoinositol Pentakisphosphate Kinase 2)	0.008	1.29
PIK3CA (phosphatidylinositol-4,5-bisphosphate 3-kinase catalytic subunit alpha)	8.22E-05	1.1
CNOT3 (CCR4-NOT Transcription Complex Subunit 3)	3.07E-06	1.08
DNMT1_fragment (DNA-methyltransferase 1)	0.004	0.961
HADH (Hydroxyacyl-CoA Dehydrogenase)	0.020	− 1.09
VIRMA (Vir Like M6A Methyltransferase Associated)	1.15E–05	− 1.13
IGKV1D-33 (Immunoglobulin Kappa Variable 1D-33)	3.40E–07	− 1.22
CYBA (Cytochrome B-245 Alpha Chain)	0.047	− 1.26
KLK4 (Kallikrein Related Peptidase 4)	0.0001	− 1.34
ERCC3 (ERCC excision repair 3, TFIIH core complex helicase subunit)	0.009	− 1.54
IKZF1 (IKAROS Family Zinc Finger 1)	0.004	− 1.85
BMP10 (Bone morphogenetic protein 10)	0.001	− 2.44
MYCBPAP (MYCBP Associated Protein)	2.81E–14	− 3.3

Adjusted p-values were calculated using the Benjamini-Hochberg method. The ratio represents a comparison of the mean abundance values of each protein between the conditions

DE analysis accentuated the distinct proteome profiles of both PE and GH versus controls (Fig. 6B). There was only 1 overlapping DE protein, haemoglobin A2 (HBA2), which was increased in both PE (*p* = 0.039, ratio = 1.28) and GH (*p* = 0.0023, ratio = 1.49), compared to control plasma.

BRCA1 interacting helicase 1 (BRIP1) (*p* = 0.046, ratio = 2.64) and diphosphoinositol pentakisphosphate kinase 2 (PPIP5K2) (*p* = 0.003, ratio = 1.66) were increased in PE compared to control plasma, suggesting a reduction in cellular ability to maintain genetic integrity and cellular homeostasis [29, 30]. An increase in pregnancy zone protein (PZP) (*p* = 3.87E-06, ratio = 1.31), an immune cell placenta-specific protein critical for successful pregnancy [31], was also observed in PE compared to control group (Fig. 6C).

ABR (ABR activator of RhoGEF and GTPase) was decreased in GH compared to control plasma (*p* = 0.043, ratio = −2.52), supporting increased regulated cell death signaling. Phosphatidylinositol-4,5-bisphosphate 3-kinase catalytic subunit alpha (PIK3CA) (*p* = 0.007, ratio = −0.871) and DNA-methyltransferase 1 (DNMT1) fragments (*p* = 0.004, ratio = −1.01) were both decreased in GH compared to control group (Fig. 6D).

The distinction between PE and GH proteomic profiles included 15 DE proteins (Fig. 6E). Cytochrome B-245 alpha chain (CYBA) (*p* = 0.047, ratio = −1.26), immunoglobulin kappa variable 1 D-33 (IGKV1D-33) (*p* = 3.40E-07, ratio = −1.22) and IKAROS family zinc finger 1 (IKZF1) (*p* = 0.004, ratio = −1.85) abundance was decreased in PE versus GH plasma, supporting a lower immune response [32]. Conversely, homeostatic proteins were increased in PE plasma, specifically phosphatidylinositol-4,5-bisphosphate 3-kinase catalytic subunit alpha (PIK3CA) (*p* = 8.22E-05, ratio = 1.1) and diphosphoinositol pentakisphosphate kinase 2 (PPIP5K2) (*p* = 0.008, ratio = 1.29), compared to GH. Another pronounced variation concerning PE versus GH was the abundance of proteins associated with the maintenance of cellular genetic material. PE plasma presented increases CCR4-NOT transcription complex subunit 3 (CNOT3) (*p* = 3.07E-06, ratio = 1.08) and DNA-methyltransferase 1 (DNMT1 fragment) (*p* = 0.004, ratio = 0.961).

## Discussion

HDP, including GH and PE, are the leading causes of mortality and morbidity in pregnancy and are associated with an increased risk of developing CVD post-partum [33]. The mechanisms of this association remain unclear, leading to the lack of effective monitoring and treatment strategies for women post-HDP. In this study, we developed and characterized 3D in vitro GH- and PE-relevant cardiac platforms to study, for the first time, cellular and molecular mechanisms of HDP five years post-partum and potentially identify early cardiac changes. We also performed a comprehensive proteomic analysis to identify potential biomarkers or therapeutic targets in the secretome that could be responsible for early signs of cardiovascular dysfunction.

Although there was no change in any particular cell type following treatment with GH plasma, there was a modest decrease in the overall proportion of live cells, whereas PE did not impact cell viability at all. This aligned with our plasma proteome profile, indicating decreased abundance of ABR, a regulator of cell death signaling [32], in GH plasma. Consequently, ABR might have implications for the advancement of CVD in GH, akin to the findings in hypertensive patients, increasing the risk of stroke and heart failure [32, 34]. Additionally, the proteomic analysis of GH revealed a notable decrease in PIK3CA abundance, a crucial component of AKT-mediated cell survival, and DNMT1, regulating cardiomyocyte gene expression, morphology, and function. The decrease in protein abundance of both PIK3CA and DNMT1 is closely linked to cell death and survival, cell morphology, and cardiac function. A reduction in the abundance of these proteins could be potentially responsible for contractile dysfunction and arrhythmia [35, 36]. Overexpression of α-SMA protein is commonly observed in the early stages of cardiac fibrosis, highly expressed in myofibroblasts, leading to increased collagen deposition and reduction in contractility of the heart through attenuated fibroblast proliferative activity [37]. Our results align with this, as we measured an increased abundance of α-SMA in CSs treated with GH-derived plasma, supporting early indications of cardiac remodeling and fibrosis in individuals five years post-GH [38]. This new insight into the mechanisms of early cardiac dysfunction post-partum associated with GH identified potential therapeutic targets that could attenuate early adverse cardiac remodeling and increased cardiac cell death signaling, potentially preventing or delaying the onset of future heart failure.

Furthermore, we demonstrated an increase in HbA2 in both GH and PE patients' plasma. This correlates with a significant rise in contraction frequency in GH- and PE-treated CSs and fractional shortening in PE-treated CSs. Previous work has shown that HbA2 is positively associated with blood pressure, and although there was no difference in blood pressure between HDP groups and healthy controls, this could indicate likely early cardiac alterations five years post-partum that could lead to hypertension in the future [39]. In contrast to normotensive patients, PE plasma presented an increase in BRIP1 expression, which is linked to genetic instability and commonly observed in women with cervical or breast cancer and postmenopausal CVD. PPIP5K2 was also increased, which could disrupt cardiac cell growth and proliferation [40]. Moreover, our analyses support an increased risk of arterial stiffness post-PE, as indicated by abnormal PZP expression, an emerging biomarker for cardiovascular risk as identified in chronic kidney disease patients [41]. This suggests that individuals five years post-PE may exhibit vascular and endothelial dysfunction indicative of early stages of CVD. The distinction between HDP plasma groups lies in the dysregulated immune response observed in PE, as supported by the measured increase in Gal-3 abundance in PE-treated CSs and a reduction in CYBA, IGKV1D-33, and IKZF1 in plasma samples. An increase in pro-inflammatory Gal-3 is linked to cardiac fibrosis and impaired angiogenesis, contributing to cardiac remodeling [42]. The biggest difference in plasma proteomes was observed between GH and PE, which could be due to the heterogeneous nature of PE. Albeit no changes in FKBPL expression were detected between groups, this could be due to the nature of the plasma, which was isolated five years after the PE-affected pregnancy, and in individuals without clinically diagnosed CVD. It is also possible that no obvious changes were evident in angiogenesis in these individuals five years post-HDP, given that no differences were demonstrated in the expression of endothelial cell marker, CD31, or vascular dysfunction marker, VE-cadherin.

In our study, we did not report on changes in cytokine profile between the groups. Cytokines such as TNF-α and IL-6 are increased in PE, and this is well-established [6]; however, whether these mediators remain elevated five years post-partum is unknown and should be explored in the future studies. We identified 20 unique differentially abundant proteins in our proteomic analysis, the expression of which should be validated in CSs in future studies alongside understanding the specific mechanisms related to each protein and how they affect cardiac function and individual cell clusters within CSs. Another potential limitation is the variable number of CSs used per group for immunofluorescence and live/dead analysis, which ranged between 20 and 36 CSs per group. This is due to different conditions (cells, gels, transfer) affecting the CS integrity; however, we had included a minimum of four CSs per patient in the analysis, which is a sufficient number of biological repeats. Finally, we used 10% plasma to treat CSs in order to ensure the viability of cells was not affected by plasma concentration, thus reducing the concentration of molecules detected by proteomic analysis. Potential confounding clinical factors include variable hormonal levels in participants, lactation status, the presence of anaemia, the gestational age of onset and the severity of PE suffered in pregnancy and fetal sex. In our study, the GH group had a significantly higher percentage of male fetal sex whereas the control and PE groups were better-matched in this respect. In terms of lactation, all participants but one included in this study, had breastfed their babies for at least 3 months as per initial enrolment information at 6 months, however the lactation status at 5 years post-pregnancy was not recorded. Therefore, larger studies are needed in the future to take into the account a number of confounding factors that can influence the risk of future CVD post-HDPs.

The findings of this study offer a novel cardiac tissue engineering platform to better understand the mechanisms of early cardiac changes regulating HDP-associated CVD risk post-partum, with the potential to be used to screen for novel biomarkers and therapeutic targets and develop personalized treatment and monitoring options for those individuals at high-risk of CVD including post-HDP. Future studies could include longer-term treatment of CSs to identify kinetics of disease progression, as well as the use of agonists and antagonists of the proteins identified as potential therapeutic targets, including αSMA and Gal-3 for the development of better treatments to prevent CVD as a consequence of HDP. Post-partum care following HDP is poorly understood, and adequate clinical management is lacking. This is a high-priority area where further research is needed to fill in the knowledge gaps and address unmet clinical needs that will improve clinical management in this high-risk population. Our findings provide a number of promising candidates as biomarkers or therapeutic targets that should be explored further and facilitate the development of proactive monitoring and intervention strategies for women post-HDP. Another strategy may include longitudinal studies tracking women who experienced HDP over extended periods (beyond five years). Our study reported results from only five patients per group albeit with multiple CSs in vitro, these experiments are currently labour-intensive and costly, therefore future studies should aim at increasing the total patient numbers, as well as consider closely patient-specific responses given the heterogeneity of HDP and the variable extracellular milieu in each individual CS. Additionally, carefully choosing the sex of cells used will help to understand the effects of sex differences on cardiac phenotype.

Future studies could generate iPSCs from the patient’s peripheral blood mononuclear cells (PBMCs) for establishing patient-specific cardiomyocytes and cardiac endothelial and fibroblast cells. These patient-specific cells could then be used to form CSs and assess differences in baseline functions and phenotype between PE, GH and healthy pregnancy groups. By pursuing these research directions, we could significantly improve the prevention, early detection, and management of CVD in women post-HDP.

## Conclusions

This study established an innovative 3D in vitro model of CVD post-HDP to unveil novel mechanistic insights into the impact of HDP secretome five years after childbirth on the heart, which is implicated in escalating the risk of long-term CVD. Our platform was able to detect early signs of cardiac dysfunction at the cellular and molecular levels despite the fact that the clinical cardiovascular and metabolic profiles of these individuals appeared healthy. Although promising, our 3D CS platform is not a direct measurement of the heart tissue of women at five years postpartum. Contractility is a promising in vitro functional assay of CSs but not directly representative of echocardiography. Nevertheless, we have showed before that the CS platform is an improved model compared to other in vitro cardiac organoid models, and that it closely recapitulates human and non-human heart biopsies [16–20]. We showed that GH and PE trigger distinct mechanistic pathways that can lead to cardiac damage and future CVD. GH activates mechanisms related to homeostasis, cell death signaling, and malfunction of cardiomyocyte contractility. In contrast, PE leads to impaired inflammatory pathways and exhibits heightened protein expression of markers associated with endothelial dysfunction. Understanding the underlying cellular and molecular processes could provide valuable insights into targeted therapeutic approaches for each condition and potentially pave the way for the implementation of personalized treatments for preventing and treating early-stage CVD following HDP.

## Supplementary Information

Supplementary Material 1.

Supplementary Material 2.

Supplementary Material 3.

Supplementary Material 4.

## Data Availability

Data are available via ProteomeXchange with identifier PXD051288.
