# Supplementary material for: 3D in vitro modelling of post-partum cardiovascular health reveals unique characteristics and signatures following hypertensive disorders in pregnancy
Source: Biol Sex Differ. 2024 Nov 25;15:94. doi: 10.1186/s13293-024-00672-6 (PMC11587612; doi:10.1186/s13293-024-00672-6)
Supplement: Supplementary file 4 — Supplementary Material 4. [file 13293_2024_672_MOESM4_ESM.docx]

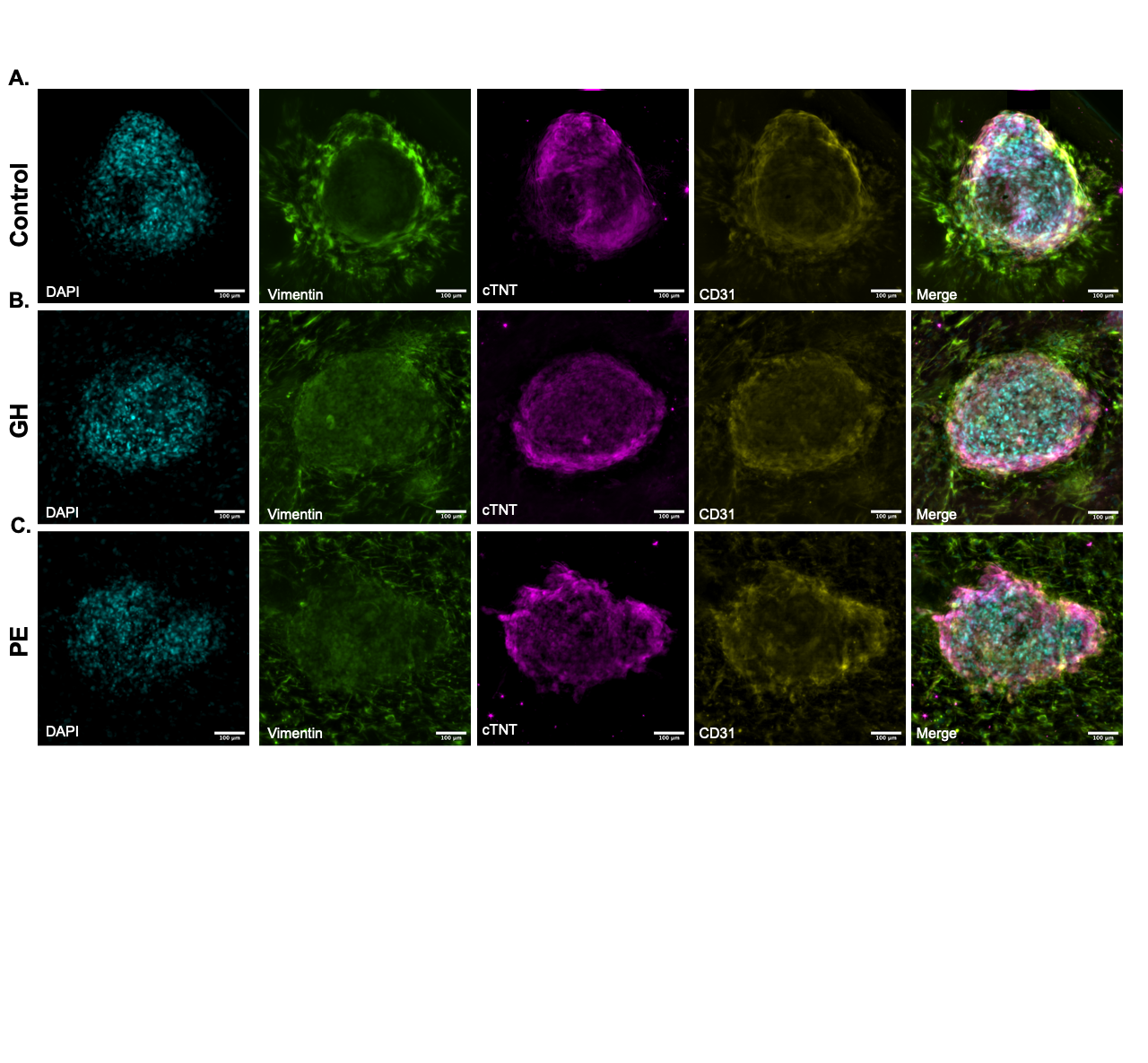
**Supplementary Figure 1. Representative images of stained cardiac spheroids with vimentin to identify cardiac fibroblasts, cardiac troponin (cTNT) for cardiomyocytes, CD31 for endothelial cells, following incubation in human plasma from healthy pregnancy (A), gestational hypertension (B) or preeclampsia (C), for 96 hours.** Images were acquired by Nikon TiE2 widefield fluorescence microscopes and the fluorescent signal intensity was quantitatively analyzed using ImageJ software. Scale bar 100µm.


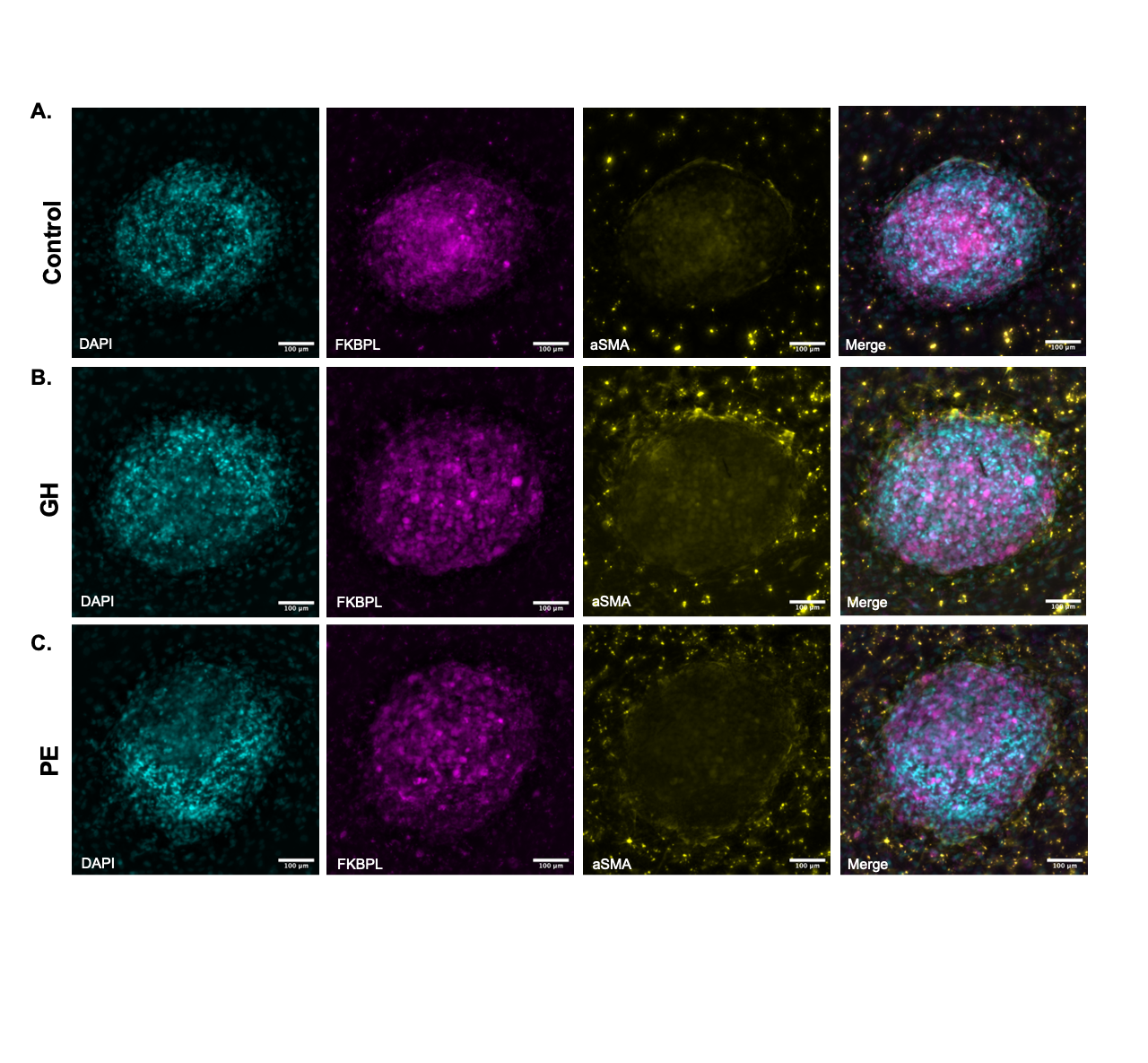


**Supplementary Figure 2. Representative images of stained cardiac spheroids with FK506-binding protein like (FKBPL) and α-SMA, following incubation in human plasma from healthy pregnancy (A), gestational hypertension (B) or preeclampsia (C), for 96 hours.** Images were acquired by Nikon TiE2 widefield fluorescence microscopes and the fluorescent signal intensity was quantitatively analyzed using ImageJ software. Scale bar 100µm.


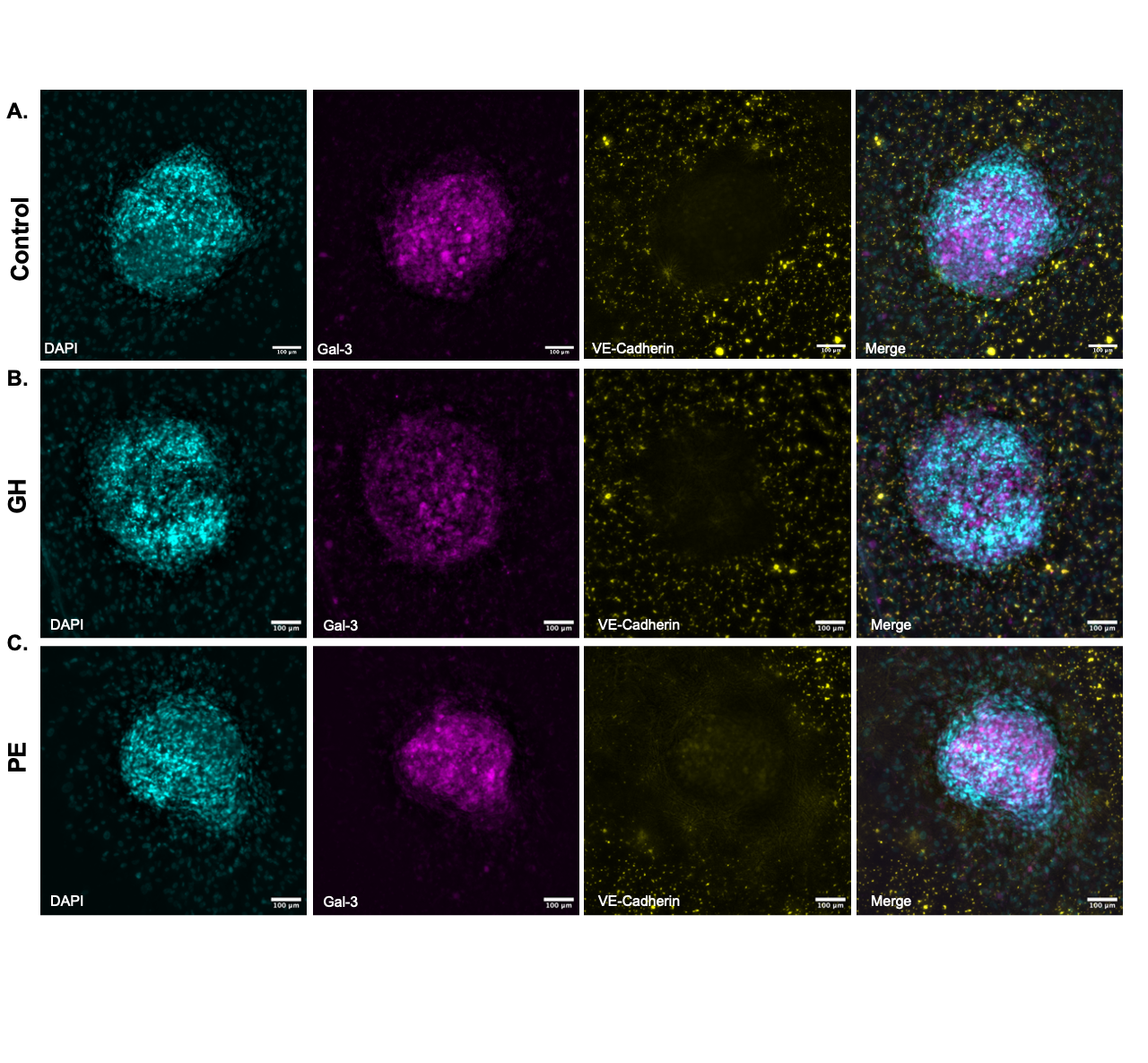


**Supplementary Figure 3. Representative images of stained cardiac spheroids with Galectin-3 (Gal-3) and VE-cadherin, following incubation in human plasma from healthy pregnancy (A), gestational hypertension (B) or preeclampsia (C), for 96 hours.** Images were acquired by Nikon TiE2 widefield fluorescence microscopes and the fluorescent signal intensity was quantitatively analyzed using ImageJ software. Scale bar 100µm.
